# Comparison of SARS-CoV-2 indirect and direct RT-qPCR detection methods

**DOI:** 10.1186/s12985-021-01574-4

**Published:** 2021-05-17

**Authors:** Joel D. Pearson, Daniel Trcka, Suying Lu, Sharon J. Hyduk, Mark Jen, Marie-Ming Aynaud, J. Javier Hernández, Philippos Peidis, Miriam Barrios-Rodiles, Kin Chan, Jim Woodgett, Tony Mazzulli, Liliana Attisano, Laurence Pelletier, Myron I. Cybulsky, Jeffrey L. Wrana, Rod Bremner

**Affiliations:** 1 Lunenfeld-Tanenbaum Research Institute, Mt Sinai Hospital, Sinai Health System, Toronto, Canada; 2 Department of Ophthalmology and Vision Science, University of Toronto, Toronto, Canada; 3 Department of Laboratory Medicine and Pathobiology, University of Toronto, Toronto, Canada; 4 Toronto General Hospital Research Institute, University Health Network, Toronto, Canada; 5 Network Collaborative Biology Centre, Lunenfeld-Tanenbaum Research Institute, Mt Sinai Hospital, Toronto, Canada; 6 Department of Molecular Genetics, University of Toronto, Toronto, Canada; 7 Department of Medical Biophysics, University of Toronto, Toronto, Canada; 8 Department of Microbiology, Sinai Health System/University Health Network, Toronto, Canada; 9 Department of Biochemistry, Donnelly Centre, University of Toronto, Toronto, Canada

**Keywords:** COVID-19, SARS-CoV-2, RT-qPCR, Direct detection

## Abstract

**Background:**

Sensitive, rapid, and accessible diagnostics continue to be critical to track the COVID-19 pandemic caused by the SARS-CoV-2 virus. RT-qPCR is the gold standard test, and comparison of methodologies and reagents, utilizing patient samples, is important to establish reliable diagnostic pipelines.

**Methods:**

Here, we assessed indirect methods that require RNA extraction with direct RT-qPCR on patient samples. Four different RNA extraction kits (Qiagen, Invitrogen, BGI and Norgen Biotek) were compared. For detection, we assessed two recently developed Taqman-based modules (BGI and Norgen Biotek), a SYBR green-based approach (NEB Luna Universal One-Step Kit) with published and newly-developed primers, and clinical results (Seegene STARMag RNA extraction system and Allplex 2019-nCoV RT-qPCR assay). We also tested and optimized direct, extraction-free detection using these RT-qPCR systems and performed a cost analysis of the different methods evaluated here.

**Results:**

Most RNA isolation procedures performed similarly, and while all RT-qPCR modules effectively detected purified viral RNA, the BGI system provided overall superior performance (lower detection limit, lower Ct values and higher sensitivity), generating comparable results to original clinical diagnostic data, and identifying samples ranging from 65 copies to 2.1 × 10^5^ copies of viral genome/μl. However, the BGI detection system is more expensive than other options tested here. With direct RT-qPCR, simply adding an RNase inhibitor greatly improved detection, without the need for any other treatments (e.g. lysis buffers or boiling). The best direct methods detected ~ 10 fold less virus than indirect methods, but this simplified approach reduced sample handling, as well as assay time and cost.

**Conclusions:**

With extracted RNA, the BGI RT-qPCR detection system exhibited superior performance over the Norgen system, matching initial clinical diagnosis with the Seegene Allplex assay. The BGI system was also suitable for direct, extraction-free analysis, providing 78.4% sensitivity. The Norgen system, however, still accurately detected samples with a clinical Ct < 33 from extracted RNA, provided significant cost savings, and was superior to SYBR green assays that exhibited reduced specificity.

**Supplementary Information:**

The online version contains supplementary material available at 10.1186/s12985-021-01574-4.

## Background

The SARS-CoV-2 coronavirus is a positive-strand RNA virus with a large genome of about 30 kb, which encodes up to 14 open reading frames, including several structural genes (e.g. Nucleocapsid (N), Spike (S), Membrane (M) and Envelope (E)), accessory genes, and a large open reading frame (Orf1a/Orf1ab) that encodes a polypeptide that is cleaved into 16 non-structural proteins [1, 2]. It is related to the SARS-CoV and MERS-CoV coronaviruses, which cause severe respiratory illness in humans, and is the causative agent of the COVID-19 respiratory disease [3]. Since the first documented case in Wuhan, China in December 2019, the virus has spread rapidly across the globe. On March 11, 2020, the WHO officially declared COVID-19 a pandemic [4, 5]. Multiple nations have experienced or are experiencing second or third waves of infection, and as of mid-April 2021 there have been over 140 million confirmed cases of COVID-19 and over 3 million deaths worldwide [6].

The wide range of disease symptoms, including a large portion of mildly or asymptomatic people, has facilitated rapid dissemination [7, 8]. Efficient diagnosis, allowing rapid and accurate patient testing remains the key to limiting disease spread. Rapid disease spread has strained the capacity of diagnostic facilities and the availability of standard reagents. The principal means of diagnostics for COVID-19 relies on RNA extraction from upper respiratory tract specimens (eg. nasal swabs) followed by reverse transcriptase-quantitative PCR (RT-qPCR) detection of viral genes (e.g. N, E and RdRp) [9–11]. Rapid development and Emergency Use Authorization (EUA) of SARS-CoV-2 RT-qPCR detection systems from many companies has helped to alleviate some of the strain by providing increased supply and alternative options to clinical diagnostic laboratories. Studies that have evaluated some kits and compared efficiency of different RT-qPCR primer sets for COVID-19 detection revealed studies large differences in sensitivity, highlighting the need for stringent comparison and further optimization of novel detection systems [12–17].

An attractive option is direct detection from patient samples without RNA extraction, as it increases throughput, decreases costs and circumvents the need for clinically approved RNA extraction reagents which have become limited. Several studies have examined the ability to directly detect patient samples collected in universal transport media (UTM). While Grant et al. [18] reported no effect on viral detection with extraction-free COVID-19 detection, several other studies noted a decrease viral detection in the order of 5–20 fold [19–24]. Interestingly, while Grant et al. [18] observed reduced detection sensitivity after heating the sample to 95 °C, others have demonstrated that heating samples to 95 °C could partially increase sensitivity [19–21], as could detergent-based lysis [21, 25]. In studies where large sample numbers were analyzed, optimized extraction-free methods resulted in a high (92–98%) concordance with clinical results, despite reduced sensitivity [19–21].

Here, we comprehensively compared two recently developed COVID-19 detection protocols, one from BGI and the other from Norgen Biotek, both of which had robust supply chains at the initiation of our studies (Table 1). The BGI system has been used extensively in several countries. The Norgen System utilizes the CDC approved N1 and N2 primer/probe sets, but a distinct, proprietary enzyme/reagent mix and is seeking approval for use starting in Canada. We compared the RNA isolation systems from both companies alongside the Qiagen RNeasy and Invitrogen Purelink systems, both of which are routinely used in research labs, and the former of which has been shown to provide only modestly reduced recovery compared to the CDC approved QIAamp Viral RNA kit [22]. We also compared and optimized BGI and Norgen Taqman RT-qPCR detection modules, as well as a SYBR green-based protocol using a commercially available RT-qPCR mix with published and newly designed primer sets. In addition, we evaluated and optimized the ability of the BGI and Norgen systems to detect SARS-CoV-2 directly from patient swabs collected in UTM without RNA extraction. Finally, we performed a cost analysis and discuss both advantages and drawbacks of the systems tested here. We observed superior performance of the BGI systems over the other systems tested, although the BGI RT-qPCR detection module was less flexible and more expensive. The BGI system provided comparable results to clinical diagnostic data, and also diagnosed patients using extraction-free detection with 78.4% sensitivity. While less sensitive, the more cost-effective Norgen RT-qPCR system still identified positive patients with clinical Ct values < 34 using extracted RNA, and direct, extraction-free detection with this system could be enhanced simply by adding an RNase inhibitor.

**Table 1:** Overview of tests used in this study

	BGI/MGI	Norgen	Qiagen RNeasy (extraction only)	SYBR Green using NEB Luna (detection only)	Seegene (clinical data)
Patient sample volume used in this study	100 μl^a^	100 μl	100 μl	N/A	300 μl
Elution volume	32 μl	32 μl	32 μl	N/A	100 μl
Target gene	Orf1ab	N (CDC N1 & N2)	N/A	S and N gene	E, N and RdRp
SARS-CoV-2 specific	Yes	Yes	N/A	Yes	Yes: N & RdRp No: E gene
Human control gene	Actin	RNase P	N/A	RNase P	None, uses PCR internal control
Criteria for positivity	CoV-2 Ct < 37 Human Ct < 35	Ct < 40	N/A	Ct < 40 with Tm matching positive control	Ct < 40

^a^Can be increased to 200 μl (manual extraction) or 160 μl (robotic extraction), but 100 μl was used in this study to match Norgen and Qiagen extraction systems

## Methods

### Patient samples

Archived nasopharyngeal swab samples were obtained from the MSH/UHN clinical diagnostics lab with approvals from the Research Ethics Boards (REB #20-0078-E) of Mount Sinai Hospital in Toronto, Canada. Samples were stored at − 80 °C, and had undergone ≥ 2 freeze–thaw cycles at the time of our analysis, with the exception of the COVID-negative sample, L013F, which was a fresh sample from the same patient as L013. Original clinical diagnostic data was obtained using the Seegene STARMag RNA extraction kit (Microlab STAR Liquid Handling System, Hamilton Company) and Allplex 2019-nCoV RT-qPCR assay analyzed using the Bio-Rad CFX96 IVD real-time qPCR detection system.

### RNA extraction

Qiagen RNeasy, Invitrogen Purelink, Norgen Biotek Total RNA Purification Kit and the BGI Magnetic Bead Viral RNA/DNA extraction kit were used as per manufacturer’s protocols. For each extraction, 100 µl of sample was used and eluted in 32 µl.

### Taqman-based RT-qPCR detection

The 2019-nCoV TaqMan RT-PCR Kit from Norgen Biotek and 2019-nCoV: Real-Time Fluorescent RT-PCR kit from BGI were used essentially as per manufacturer’s instructions. Ct value cut-offs used to determine positive versus negative samples were as per the manufacturer’s protocol (Table 1). For comparison of different plate formats (Additional file 1: Fig. S1a), 10 and 20 µl reaction volumes were used with either 2.5 or 5 µl synthetic RNA standard (Twist Biosci.), respectively, using the Norgen system. These were analyzed in parallel on separate BioRad CFX96 (20 μl reactions) or CFX384 (10 μl reactions) real-time PCR systems. All other experiments used 10 µl reaction volumes (384-well plates) with 2.5 µl of sample (synthetic standard, extracted RNA or direct UTM) and were analyzed using a Bio-Rad CFX384 detection system. For testing alternative primers/probes with the Norgen system, primers/probes were purchased from Integrated DNA Technologies (IDT) and primers were used at 500 nM with probes at 250 nM. Probes were FAM-labelled, E Sarbeco and HKU Orf1 sequences are published [12, 16], and newly designed N gene primers/probe (N Pearson) sequences are Fwd: CCAGAATGGAGAACGCAGT, Rev: TGAGAGCGGTGAACCAAGA, probe: GCGATCAAAACAACGTCGGCCCC). RT-qPCR cycling protocols were as per manufacturers recommendations, except for the testing of alternative annealing/elongation temperatures (Additional file 1: Fig. S1f) with the Norgen system where the indicated temperatures were used.

### SYBR green RT-qPCR detection

Primer pairs were designed using PrimerQuest software, and purchased from IDT. Primers selected for testing had ΔG values for self-dimers and heterodimers greater than − 9.0 kcal/mole. Newly designed primers were specific for SARS-CoV-2 with no cross-reactivity to other coronaviruses based on published sequences (SH N1 Fwd: AATTGCACAATTTGCCCCCA, Rev: ACCTGTGTAGGTCAACCACG; SH S1 Fwd: TCAGACAAATCGCTCCAGGG, Rev: TCCAAGCTATAACGCAGCCT). The published S gene primers used in this study were S1 Fwd: CCTACTAAATTAAATGATCTCTGCTTTACT, Rev: CAAGCTATAACGCAGCCTGTA [26]. Primers were used at 400 nM. RT-qPCR was performed on a LightCycler 480 (Roche) with a 384 well plate using the NEB Luna Universal One-Step RT-qPCR kit (NEB #E3005L, New England Biolabs Inc) and a reaction volume of 10 μl with 2.5 μl of sample. Cycling conditions were as follows: 55 °C for 10 min (RT), 95 °C for 1 min (denaturation), 45 cycles: 95 °C for 10 s, 60 °C for 30 s (amplification), melt curve. Standard curves were generated for each primer set with serial dilutions of viral RNA from 0.8 to 800,000 copies/μl; SARS-CoV-2 RNA (strain USA_WA1/2020) was provided by the World Reference Centre for Emerging Viruses and Arboviruses (Galveston, TX) (WRCEVA).

### Direct extraction-free SARS-CoV-2 detection

For direct detection, 2.5 μl of patient sample in UTM (Copan) were added to the RT-qPCR reaction mix. For comparison to extracted RNA, an equivalent input of extracted RNA was used (i.e. extracted RNA eluted in 32 μl was diluted 1:2 with RNase-free water). To optimize direct detection, RNaseOUT (ThermoFisher) was added to UTM samples (2 U/μl). Samples were then left untreated, heated at 95 °C for 15 min, mixed 1:1 with Lucigen QuickExtract DNA extraction solution with heating at 95 °C for 5 min or treated with MyPOLS Bio VolcanoCell2G lysis buffer, 1% Triton X-100, 1% Tween-20 or 1% Saponin and incubated on ice 15 min. Samples were then directed added to the RT-qPCR reaction mixture and compared to UTM samples that had been left untreated.

### Statistical analysis

Receiver Operator Characteristic (ROC) curve analysis was performed using MedCalc software according to methodology by DeLong et al. [27]. Liddell’s test and confidence intervals for sensitivity and specificity calculations were determined using StatsDirect v3 software. Paired, 2-tailed t-tests used to compare Ct values were calculated using GraphPad Prism software.

## Results

### Comparison of RNA extraction and RT-qPCR detection methods

Many diagnostic protocols utilize 20 µl reactions in 96-well plates. Using the Norgen RT-qPCR COVID-19 detection kit (which utilizes CDC-approved N1 and N2 primers), we observed similar Ct values in a comparison of 20 versus 10 µl reactions in 96- or 384-well plates, respectively (Additional file 1: Fig. S1a). Thus, to reduce cost and increase throughput we focused on 384-well plates.

**Fig. 1:**
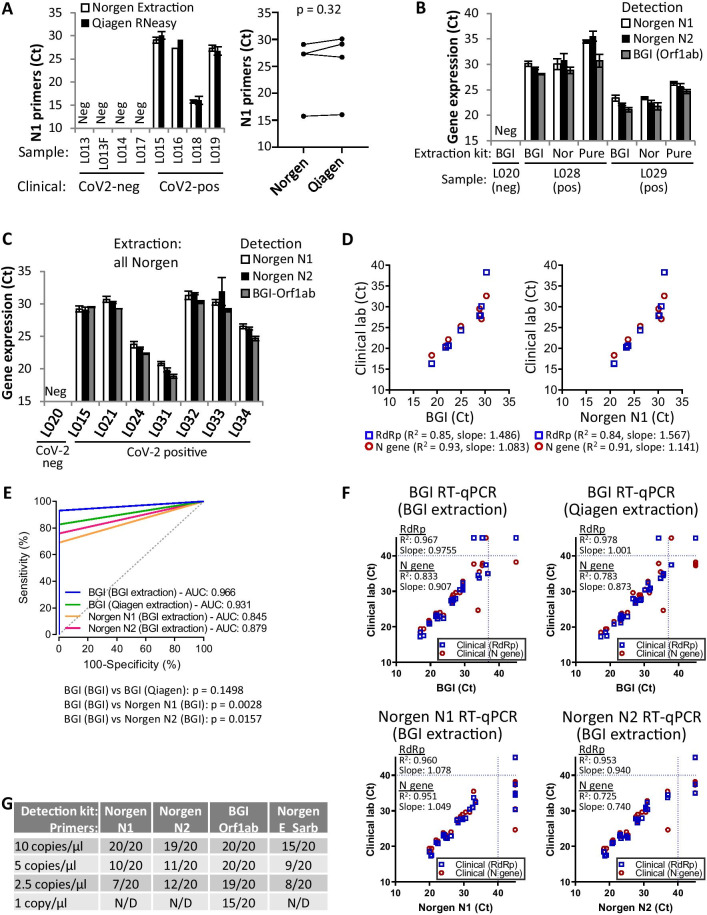
BGI detection kit shows superior performance over the Norgen kit. **a** Analysis of four negative and four positive patient samples extracted with either the Qiagen RNeasy or Norgen RNA isolation kits using the Norgen RT-qPCR detection system with N1 primer/probe sets. Samples L015, L018 and L019 are the mean ± range of technical duplicates run independently on two separate plates, other samples were analyzed once. A paired t-test was used to compare Norgen versus Qiagen extractions. **b** Analysis of two positive patient samples extracted with the BGI, Norgen (Nor) or Invitrogen Purelink (Pure) RNA isolation kits using the Norgen (N1 or N2 primers/probes) or BGI RT-qPCR detection systems. Mean ± std dev of the same sample run on three (BGI & Norgen extractions) or two (Purelink extraction) separate plates. **c** Analysis of additional patient samples using the Norgen (N1 or N2 primers) or BGI detection systems. Mean ± range of the same samples run independently on two separate plates. **d** Comparison of Ct values from original clinical diagnosis (Seegene Allplex RdRp and N genes) and data obtained with the BGI or Norgen detection systems. **e** ROC curves comparing performance of BGI versus Qiagen RNeasy extraction kits and BGI versus Norgen RT-qPCR detection kits on larger cohort of 29 positive and 30 negative samples. n/a: not applicable. **f** Comparison of Ct values from original clinical diagnosis (Seegene Allplex RdRp and N genes) and data obtained with the BGI or Norgen detection systems in the larger cohort of 29 positive samples. Horizontal dotted line indicates Ct threshold for positive clinical detection and vertical dotted line indicates Ct threshold for positive detection using the BGI or Norgen systems. Note, that R^2^ and slope calculations include only samples that were detected with Ct < 40. **g** Detection limit determination using the BGI or Norgen detection systems shown as the number of positives/total number of wells. Concentrations are in copies/µl in the standard. N/D: not determined

We assessed four extraction methods. First, we compared the widely used Qiagen RNeasy RNA extraction kit to another column-based kit from Norgen Biotek. None of the SARS-CoV-2-negative samples generated any signal, and we detected no significant difference in Ct values across four clinically-diagnosed positive patient samples (Fig. 1a and Additional file 1: S1b), suggesting similar extraction efficiencies of these two systems. We next compared efficiency of the Norgen (column-based), Invitrogen Purelink (column-based) and BGI (magnetic bead-based) RNA isolation systems. We tested each of these three extraction methods with three recently developed Taqman detection systems, including Norgen N1 and N2 primers, plus BGI primers targeting the Orf1ab gene. Ct values for two new positive patient samples were similar with both the Norgen and BGI extraction systems, but higher with the Invitrogen kit, independent of detection method (Fig. 1b). For all three extraction methods, the BGI RT-qPCR system demonstrated Ct values ~ 1–3 cycles better than either of the Norgen primers (Fig. 1b), and similar results were obtained with seven additional samples all isolated using the Norgen RNA extraction kit (Fig. 1c). Pairwise analysis confirmed a statistically significant improvement with the BGI primers (Additional file 1: Fig. S1c). Original clinical Ct values were available for 8/9 of these samples, which were obtained using the Seegene STARMag RNA extraction kit and Allplex 2019-nCoV RT-qPCR assay targeting the SARS-CoV-2N and RdRp genes. We observed a strong correlation between the clinical data and Ct values obtained using either the BGI or Norgen RT-qPCR detection modules (Fig. 1d and Additional file 1: Fig. S1d). Notably, there was no significant difference between the BGI and the clinical RdRP or N gene Ct values (Additional file 1: Fig. S1d). While the Ct values obtained with the N1 and N2 Norgen primers were not significantly different from the clinical RdRP Ct values, both were significantly higher than the clinical N gene values (Additional file 1: Fig S1d). In summary, these initial analyses suggested that the BGI system exhibits better performance than the Norgen system.

To validate these data, we utilized a larger patient cohort of 59 clinically-diagnosed samples (29 positive and 30 negative). Both the Qiagen RNeasy and BGI extraction methods (using BGI RT-qPCR detection) demonstrated 100% specificity on this larger cohort, and while sensitivity was slightly higher with the BGI extraction system (93.1% vs. 82.7%, Additional file 1: Fig. S1e), this difference was not significant. We then compared the BGI and Norgen RT-qPCR detection modules using the BGI-extracted samples. While both provided 100% specificity, the 93.1% sensitivity with BGI detection system outperformed the 69.0% or 75.9% sensitivities with Norgen N1 or N2 primers, respectively, although only the difference between BGI and Norgen N1 primers was significant (Additional file 1: Fig. S1e). It is important to note that for a positive diagnosis, the Norgen system requires detection with both the N1 and N2 primers/probes, so sensitivity will be dictated by the primers with poorer performance (in this case for the N1 gene). The human control gene was detected in all samples tested (not shown). Similar to these findings, area under the curve (AUC) data from Receiver Operator Characteristic (ROC) curve analyses confirmed that the BGI RT-qPCR system significantly outperformed the Norgen N1 and N2 primers (p < 0.05 for both, Fig. 1e). As with the pilot cohort (Fig. 1d), Ct values with the BGI, or Norgen N1 and N2 primers correlated strongly to those obtained for RdRp and N gene at original clinical diagnosis (Fig. 1f). As before (Fig. 1d), BGI and clinical Ct values were comparable, whereas Norgen Ct values were higher (Fig. 1f). The Norgen detection system performed well on samples with clinical values of Ct < 34 (20/21 positives detected, with 1/21 inconclusive as only N2 primers were positive), but not on those with Ct > 34 (0/8 positives detected with 1/8 inconclusive in which only N2 primers were positive) (Fig. 1f). In contrast, the BGI system performed well across the entire range of clinical Ct values, and of the 29 clinical positives tested, the two “false negatives” were actually marginal/ambiguous clinical positives with very high Ct values (> 38) for the N gene and negative for both the RdRp gene (Fig. 1f) and the pan-Sarbecovirus E gene (not shown). Whether this result was affected by RNA degradation due to freeze–thaw of the samples is unknown, but remains a possibility.

Next, we compared the limit of detection (LOD) of the BGI and Norgen RT-qPCR systems. For this, we ran 20 replicates each with various concentrations of synthetic SARS-CoV-2 standards (TWIST Bioscience), with LOD defined as the concentration exhibiting ≥ 95% (19/20) sensitivity. The LOD with BGI primers was 2.5 copies/μl, compared to 10 copies/μl with the Norgen primers (Fig. 1g). To determine if the latter could be enhanced, we tested different annealing/elongation temperatures in the qPCR reaction along with two other published SARS-CoV-2 primers/probes shown to have high sensitivity (E Sarbeco and HKU Orf1) [12, 16, 28, 29], and new primers/probes we designed to target the viral N gene. The recommended annealing/elongation temperature for the Norgen system is 55 °C whereas the BGI system utilizes 60 °C, but increasing the temperature did not affect Ct values for either the N1 or N2 primers (Additional file 1: Fig. S1f). Using the Norgen RT-qPCR mix, we observed poor performance of the HKU Orf1 primer set, and the newly designed N gene primers provided higher Ct values compared to the CDC N1 and N2 primers, but the E gene primers/probes demonstrated lower Ct values compared to the N1/N2 primers, particularly at an annealing/elongation temperature of 59 °C (Additional file 1: Fig. S1f). This improvement, however, did not translate to a better LOD (Fig. 1g). Thus, while both BGI and Norgen detection systems reliably detect purified SARS-CoV-2 RNA from patients with clinical values Ct < 34, the BGI detection system provides a lower LOD and higher sensitivity.

### SYBR green detection

**Fig. 2:**
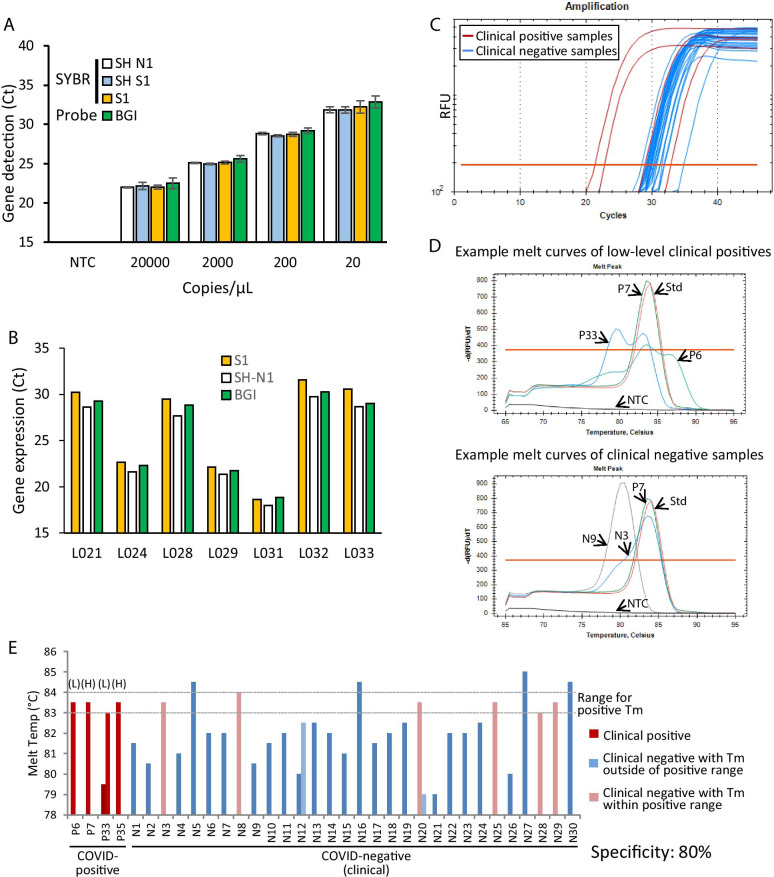
SYBR green detection of extracted RNA. **a** Serial dilutions of SARS-CoV-2 synthetic RNA standards (Twist Biosci) were run in SYBR green and BGI TaqMan assays. Mean ± STD; n ≥ 3. **b** Comparison of Ct values obtained for each patient sample with the SYBR green and BGI TaqMan assays. BGI data is from Fig. 1b, c. **c** Amplification cures for four additional positive and 30 negative patient samples using SYBR green and the SH-N1 primers. **d** Example melt curves from a synthetic Twist SARS-CoV-2 RNA standard (std), high-level positive sample (P7) or two low-level positive samples (P6 and P33) (top) or two negative patient samples (N3 and N9) (bottom). **e** Melt temperatures (Tm) of the two high-level positive samples (H, P7 and P35), two low-level positive samples (L, P6 and P33) and 30 clinical negative samples (N1–N30). Dotted lines indicate acceptable range for a positive Tm (synthetic SARS-CoV-2 RNA ± 0.5 °C)

Next, we compared the BGI detection system to a SYBR green-based method. We tested various published primers, some designed for SYBR green and some from TaqMan assays [12, 16, 26, 30], and designed our own. One published set for the viral S gene [26] and two new N or S gene primer sets gave little/no signal in no-template control (NTC) samples and generated a linear response across 8—800,000 viral copies/μl (unpublished observation). These were thus selected for future analyses. We then compared SARS-CoV-2 standards using the SYBR green primers and the BGI detection kit and observed comparable Ct values between the two systems across 20 to 20,000 genome copies/μl (Fig. 2a). Identical Ct values were obtained using SARS-CoV-2 RNA from WRCEVA (unpublished observation). The BGI system provided a slightly better LOD than the SYBR green systems (compare Fig. 1g and Additional file 1: S2a).

We next analyzed a pilot cohort of 7 positive and 2 negative patient samples comparing the SYBR green primers to previous data obtained with the BGI kit (Fig. 2b and Additional file 1: Fig. S2b). One of the primers (SH S1) did not perform well on patient samples and was excluded from these experiments. The other SYBR green primers reliably identified all 7 positive patient samples, with SH-N1 primers generating slightly lower Ct values (0.3 to 1.1 Ct values, p = 0.02) and S1 primers providing slightly higher Ct values compared to the BGI system (− 0.2 to 1.6 Ct values, p < 0.01). Quantification of viral gene copy numbers generated similar results for SYBR green and BGI, and ranged from 24 copies to > 120,000 copies/µl (Additional file 1: Fig. S2c). Melt curve analysis revealed non-specific amplification in the small number of negative and low virus copy positive samples in this cohort (Additional file 1: Fig. S2d). Therefore, we analyzed 2 additional higher-level positive patient samples (P7 and P35), 2 low-level positive samples (P6 and P33) and, to rigorously assess specificity, 30 negative clinical samples. While higher-level positives were easily identified, we observed amplification in all 30 clinical negative samples, which gave similar Ct values as low-level positives (Fig. 2c). Melt curve analysis demonstrated that high-level positive samples (P7, P35) gave specific melt peaks comparable to synthetic SARS-CoV-2 standards, while low-level samples (P6, P33) gave multiple melt peaks, one of which overlapped with standards (Fig. 2d,e). Among the 30 negatives, the melting temperatures of 24 were distinct from the synthetic SARS-CoV-2 standards, but 6 matched those of positive samples (Fig. 2d,e), indicating a specificity of only 80%. Thus, with the primers and conditions tested, SYBR green detection generated several false-positives, even with melt curve analysis.

### One step detection without RNA purification

**Fig. 3:**
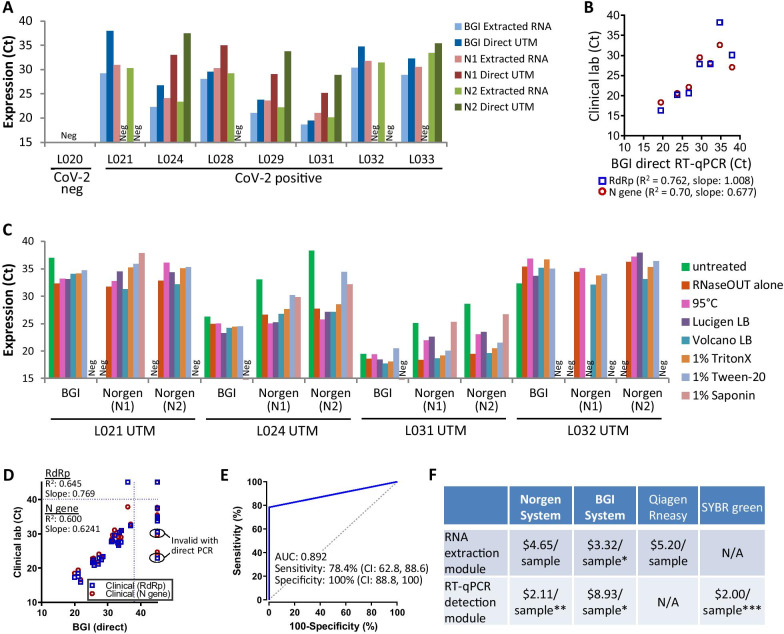
One-step direct detection without RNA extraction. **a** Analysis of extracted RNA or direct UTM from a panel of patients using the BGI or Norgen (N1 and N2 primers) detection systems. **b** Comparison of Ct values from original clinical diagnosis with extracted RNA (Seegene Allplex RdRp and N genes) to data obtained for direct analysis with the BGI detection system. **c** Patient samples in UTM were left untreated, or treated with the RNase inhibitor RNaseOUT with or without heating at 95 °C for 15 min, or treated with the indicated lysis buffers/detergents and then directly analyzed using the BGI or Norgen (N1/N2 primers) RT-qPCR detection systems. Note sample L020 (clinical negative) was also tested under these conditions and was confirmed as SARS-CoV-2 negative. **d** Comparison of Ct values from original clinical diagnosis (Seegene Allplex RdRp and N genes) and data obtained with the BGI or Norgen detection systems in the larger cohort of 30 positive samples. Horizontal dotted line indicates Ct threshold for positive clinical detection and vertical dotted line indicates Ct threshold for positive detection using the BGI system. Note, that R^2^ and slope calculations include only samples that were detected with Ct < 40. **e** ROC curve for direct analysis using the BGI RT-qPCR detection system including all 37 positive and 31 negative sample analyzed. **f** Cost analysis comparing Norgen, BGI, Qiagen and SYBR green systems. Price is in USD at the time these studies were initiated (late March/early April 2020) for 10 µl RT-qPCR reactions and include relevant processing and shipping fees, and would be influenced by regional pricing. *BGI RNA extraction module is based on the 96-sample format, price can be reduced ~ 15% by purchasing the 1728-sample format, and bulk pricing with a ~ 25% discount of the detection module is available for > 10,000 samples. **Pricing for the Norgen detection module is based on the 50-sample format running three separate wells (N1, N2 and RNaseP) per sample, pricing can be reduced if purchasing the larger 500-sample format. ***Pricing for SYBR green detection is based on the 200 reaction size LUNA Universal One-Step RT-qPCR Kit (NEB) running three separate wells/sample (two viral genes and one human control gene). Pricing can be reduced up to 30% with larger kit sizes. N/A: not applicable

To reduce the number of steps required for viral detection, we tested direct, extraction-free RT-qPCR on patient samples in UTM. For this, we added 2.5 μl of sample directly to the RT-qPCR mix and compared this to an equivalent input of extracted RNA. UTM blocked SYBR-green detection of SARS-CoV-2 RNA standards (unpublished observation), but both the BGI and Norgen TaqMan detection systems identified positive patient samples (Fig. 3a). Ct values were lower for BGI *vs* Norgen, consistent with data from purified RNA (c.f. Fig. 1c and Additional file 1: S1c to Fig. 3a). Furthermore, the Norgen system did not reliably identify some positive samples with lower levels of virus (Fig. 3a). Relative to extracted RNA, virus detection by direct RT-qPCR with the BGI detection kit was 2–26 fold lower as assessed by quantification of viral copy number (except sample L021, which was ~ 600-fold reduced, see below for an explanation), whereas with the Norgen kit it was 20–1000 fold lower with direct detection (L033 with the N2 primers was an exception at 4.4-fold). Despite the higher Ct values, there was a strong correlation between BGI and original clinical Ct values (Fig. 3b).

Others reported that heat or different lysis buffers/detergents may improve direct detection [19–21, 25]. Thus, using a pilot series of four patient samples, we assessed the effect of heating at 95 °C for 15 min, five different lysis buffers/detergents (Lucigen QuickExtract DNA extraction solution, MyPOLS Bio VolcanoCell2G lysis buffer, 1% Triton X-100, 1% Tween-20 or 1% Saponin), and treatment with the RNase inhibitor, RNaseOUT. Notably, simply adding the RNase inhibitor was sufficient to dramatically increase virus detection > 100 fold (as assessed by copy number) using the Norgen system, and generated Ct values comparable to those obtained with the BGI RT-qPCR system. Most importantly, this permitted detection of previous “false-negative” samples L021 and L032 (Fig. 3c). Furthermore, addition of the RNase inhibitor brought direct RT-qPCR results with the Norgen detection kit to within 3 Ct values (~ 10 fold) of those obtained with extracted RNA (compare Fig. 3a, c). Treatment with heat, lysis buffers or detergents did not appreciably increase virus detection, and in some cases reduced virus detection (higher Ct values). For the BGI detection system, none of the treatments dramatically improved detection, with the exception of sample L021 (Fig. 3c), which previously showed the largest difference between extracted RNA and direct UTM analysis (Fig. 3a). We presume, therefore, that L021 had higher RNase levels that were not fully inhibited by the (proprietary) RNase inhibitor already present in the BGI mix. Thus, addition of an RNase inhibitor is sufficient to improve direct detection, and under these conditions BGI and Norgen kits perform similarly.

Following these pilot assays, we assessed direct detection on 60 patient samples, including 30 clinical positives and 30 negatives, focusing on the more sensitive BGI detection system. We observed a strong correlation with clinical Ct values, and, with the exception of two invalid samples in the direct RT-qPCR (no human actin detected), accurately identified all samples with original clinical Ct values < 33 (Fig. 3d). Akin to our observation with most samples in the pilot study, adding RNaseOUT did not further improve direct detection (unpublished observation). Combining the 31 negatives and 37 positives from the pilot and expanded datasets (Fig. 3a, d), the BGI direct detection strategy generated an AUC of 0.892 in ROC curve analysis, and this test exhibited a sensitivity of 78.4% and specificity of 100% (Fig. 3e).

## Discussion

Here, we compared four different RNA isolation methods, two recently released SARS-CoV-2 TaqMan RT-qPCR detection modules and a SYBR green-based RT-qPCR approach for SARS-CoV-2 detection using published and newly-developed primers. In addition, we tested and optimized extraction-free SARS-CoV-2 detection. Overall, we found that the BGI extraction and detection system provides excellent specificity and sensitivity with either extracted RNA or raw patient samples.

In all our assays we favored 10 μl 384-well versus 20 μl 96-well reactions, to reduce cost and increase throughput. Sample pipetting errors may increase with smaller wells, although that can be avoided/minimized using multi-channel pipettes or robotics, which may require more highly trained personnel. For RNA extraction, we tested three different column-based systems from Qiagen (RNeasy), Invitrogen (Purelink) and Norgen Biotek, as well as a magnetic bead system from BGI. While only tested on a small sample set, we observed similar results using the Norgen and BGI systems, but lower recovery of viral RNA with the Invitrogen Purelink system. Analysis with an expanded cohort of patient samples revealed that the BGI extraction protocol provides superior sensitivity over Qiagen RNeasy columns. According to Canadian pricing, the Norgen Biotek RNA isolation system is ~ 40% more expensive than that of BGI ($4.65 vs. $3.32/sample), while the Qiagen RNeasy was even more expensive ($5.20/sample, Fig. 3f), but we found that for small sample batches the bead-based BGI kit was slower, increasing sample preparation time by about 50% over the Norgen or Qiagen kits (~ 30 vs. 45 min). This difference was largely due to two incubation steps in the BGI protocol, so the relative difference in sample preparation time may diminish with larger numbers. Furthermore, magnetic beads facilitate large-scale, automated sample extraction. Given the cost savings (vs. Norgen and Qiagen) and superior performance (vs. Qiagen) of the BGI extraction system, it provides several advantages over the other systems.

For RNA detection, we tested TaqMan-based detection systems from BGI and Norgen Biotek, as well as a SYBR green method using a commercially available RT-qPCR mix and published primers (some used for SYBR green and others from probe-based methods) along with new primers we developed. While all systems could accurately detect SARS-CoV-2 positive patient samples at lower Ct values (higher viral titres) using extracted RNA, and generated Ct values that strongly correlated with clinical diagnostic values, the BGI system provided superior performance over Norgen (lower Ct values, lower LOD, higher sensitivity and a larger AUC from ROC curve analysis). While we did not rigorously assess sensitivity of the SYBR green system with a large series of positive samples, analysis of an extensive series of negative clinical samples exposed significant specificity problems. We also tested 8 other published and newly designed primers and all yielded non-specific PCR products (unpublished observation). Whether non-specific products can be eliminated using alternative RT-qPCR mixes remains to be determined.

The major drawback of the BGI detection module is the higher cost, as it is over four-times more expensive than the Norgen or SYBR green methods (Fig. 3f). Cost savings with the Norgen kit could be even greater if multiplexing primers/probes were utilized. The Norgen system follows CDC guidelines with three separate reactions, one each using FAM-labelled viral N1, viral N2 or human RNase P primers/probes. The Norgen system also provides more flexibility than BGI as the primers/probes come pre-mixed in the latter and cannot be altered, whereas they are added separately in the Norgen system, allowing alternative primer/probe options and concentrations. We tested three alternative primers/probes with the Norgen system. Those targeting the E gene performed similarly to the provided N1/N2 primers/probes, while alternatives for the viral N or Orf1a gene performed poorly, although only a single primer/probe concentration was tested. The BGI primers/probe targets the Orf1a gene, but exact sequences are unavailable, and only a single primer/probe set is used. Mutations in this single target could thus affect detection and generate false negatives. This is of increasing concern as new variants of SARS-CoV-2 emerge, although BGI has announced (https://www.bgi.com/us/wp-content/uploads/sites/2/2021/01/RT-PCR-Performance-Notification-011321.pdf) that their primers are unaffected by mutations in the recently described B.1.1.7 UK variant [31], which shows greater rates of infectivity, or the 501Y.V2 South African variant [32], which may evade spike protein antibodies raised against earlier variants and/or the vaccines currently being deployed around the world. Overall, the BGI system provides greater sensitivity, but the Norgen system offers greater flexibility and reduced costs. Patients with low viral loads are less infectious, and several studies suggest that while patients with Ct values ≤ 25 are likely to be infectious, those with clinical Ct values above 33–34 are not [33–35]**.** We found that the Norgen system identified 20/21 positive samples with a clinical Ct < 34 (95.2% sensitivity), so it may be acceptable in certain settings given the financial savings.

Finally, we tested direct, extraction-free detection of SARS-CoV-2. This approach reduces cost, increases throughput, and circumvents the need for RNA extraction systems that may be scarce during a pandemic. Others have shown that SARS-CoV-2 can be detected from patient samples, although this typically comes with reduced virus detection, which can at least partially be overcome by heat and/or detergent lysis [19–21, 25]. We found that SYBR green-based detection was incompatible with direct detection of samples in UTM. The unmodified BGI detection system performed well in the direct detection of unprocessed patient samples (78% sensitivity), and confirmed most positive samples (except two that were scored as “invalid”) with clinical Ct values < 34. The Norgen system initially performed poorly on direct UTM samples, generating much higher Ct values than extracted RNA (in some cases 1000 s of fold higher), and resulted in several false-negatives. However, adding an RNase inhibitor increased virus detection using direct RT-qPCR with the Norgen system > 100-fold, allowing detection of all previously false-negative samples. This came with an added cost of ~ $0.61 (USD)/sample, significantly less than the cost of RNA extraction and with much reduced time. Other strategies have been used to minimize RNase contamination [36]. This modification did not, in most cases, dramatically affect direct sample analysis with the BGI detection system, suggesting it already contains an RNase inhibitor. Even in that case however, detection of one patient sample was markedly improved, implying higher RNase levels. Thus, addition of an RNase inhibitor is a simple and sufficient step to facilitate diagnosis of SARS-CoV-2 directly from patient samples.

## Conclusions

Our results provide an in depth analysis of recently released SARS-CoV-2 detection systems from BGI and Norgen Biotek, and compare these to a SYBR green-based approach and to clinical diagnostic values. Overall, we found that the BGI RT-qPCR system provided superior performance, while the Norgen system provided satisfactory sensitivity at lower cost and greater flexibility, but we encountered major specificity issues with SYBR green based detection. These findings will help guide selection of SARS-CoV-2 detection systems and provide a template for comparison with alternative systems.

## Supplementary Information

**Additional file 1: Fig. S1.** BGI detection kit shows enhanced sensitivity over Norgen kit. (**A**) Serial dilutions of SARS-CoV-2 synthetic RNA standards from Twist Biosci (in copies/μl of the standard added to the RT-qPCR reaction) run in parallel on separate BioRad CFX 96-well (20 μl reactions) or 384-well (10 μl reactions) real-time PCR systems using the Norgen COVID-19 RT-qPCR detection module. Mean +/− range of two independent tests. (**B**) Analysis of four negative and four positive patient samples extracted with either the Qiagen RNeasy or Norgen RNA isolation kits using the Norgen RT-qPCR detection system with N2 primer/probe sets. Samples L015, L018 and L019 are the mean +/− range of technical duplicates run independently on two separate plates, other samples were analyzed once. A paired t-test was used to compare Norgen vs. Qiagen extractions. (**C**) Pairwise comparison of Ct values obtained with BGI vs. Norgen (N1 and N2 primers/probes) RT-qPCR detection systems. Paired t-tests were used to compare results. (**D**) Comparison of Ct values from original clinical diagnosis (Seegene Allplex RdRp and N genes) and data obtained with the BGI or Norgen detection systems. Paired t-tests were used to compare results. (**E**) Sensitivity and specificity of BGI vs. Qiagen RNeasy extraction kits and BGI vs. Norgen RT-qPCR detection systems. (**F**) Analysis of 500 viral copies (Twist Biosci) using N1, N2, E Sarbeco, HKU Orf1 and our N gene (N_Pearson) and the Norgen RT-qPCR mix with the indicated annealing/elongation temperatures. Mean +/− range of two independent tests. **Fig. S2** SYBR green detection of SARS-CoV-2. (**A**) Detection limit for each of the SYBR green primer sets shown as the number of positive samples/total number of samples tested. Synthetic RNA (Twist Biosci) was used from stocks with the indicated number of copies per μL. (**B**) Comparison of Ct values obtained for each patient sample with the SYBR green and BGI TaqMan assays. Linear regression was used to determine the R2. BGI data is from Fig. 1b and c. (**C**) Comparison of viral copy number per μL for each of the positive patient samples determined with each primer set. Copy number was determined using a standard curve of SARS-CoV-2 RNA. (**D**) Examples of melt curves from a positive high SARS-CoV-2 copy number sample (L024) showing a single specific melt peak, a negative sample (L017) showing non-specific melt peaks, and a positive low SARS-CoV-2 copy number sample (L032) showing both specific and non-specific melt peaks. NTC, no template control (water).

## Data Availability

Data sharing is not applicable to this article as no datasets were generated or analysed during the current study.

## Abbreviations

EUA: Emergency Use Authorization
NTC: No template control
RT-qPCR: Reverse transcriptase-quantitative PCR
UTM: Universal transport media
WRCEVA: World Reference Centre for Emerging Viruses and Arboviruses
