# Supplementary material for: Comparison of SARS-CoV-2 indirect and direct RT-qPCR detection methods
Source: Virol J. 2021 May 17;18:99. doi: 10.1186/s12985-021-01574-4 (PMC8127261; doi:10.1186/s12985-021-01574-4)
Supplement: Supplementary file 1 — Additional file 1: Fig. S1. BGI detection kit shows enhanced sensitivity over Norgen kit. (A) Serial dilutions of SARS-CoV-2 synthetic RNA standards from Twist Biosci (in copies/μl of the standard added to the RT-qPCR reaction) run in parallel on separate BioRad CFX 96-well (20 μl reactions) or 384-well (10 μl reactions) real-time PCR systems using the Norgen COVID-19 RT-qPCR detection module. Mean +/− range of two independent tests. (B) Analysis of four negative and four positive patient samples extracted with either the Qiagen RNeasy or Norgen RNA isolation kits using the Norgen RT-qPCR detection system with N2 primer/probe sets. Samples L015, L018 and L019 are the mean +/− range of technical duplicates run independently on two separate plates, other samples were analyzed once. A paired t-test was used to compare Norgen vs. Qiagen extractions. (C) Pairwise comparison of Ct values obtained with BGI vs. Norgen (N1 and N2 primers/probes) RT-qPCR detection systems. Paired t-tests were used to compare results. (D) Comparison of Ct values from original clinical diagnosis (Seegene Allplex RdRp and N genes) and data obtained with the BGI or Norgen detection systems. Paired t-tests were used to compare results. (E) Sensitivity and specificity of BGI vs. Qiagen RNeasy extraction kits and BGI vs. Norgen RT-qPCR detection systems. (F) Analysis of 500 viral copies (Twist Biosci) using N1, N2, E Sarbeco, HKU Orf1 and our N gene (N_Pearson) and the Norgen RT-qPCR mix with the indicated annealing/elongation temperatures. Mean +/− range of two independent tests. Fig. S2 SYBR green detection of SARS-CoV-2. (A) Detection limit for each of the SYBR green primer sets shown as the number of positive samples/total number of samples tested. Synthetic RNA (Twist Biosci) was used from stocks with the indicated number of copies per μL. (B) Comparison of Ct values obtained for each patient sample with the SYBR green and BGI TaqMan assays. Linear regression was used to determine the R2. BGI d [file 12985_2021_1574_MOESM1_ESM.pptx]

## Slide 1
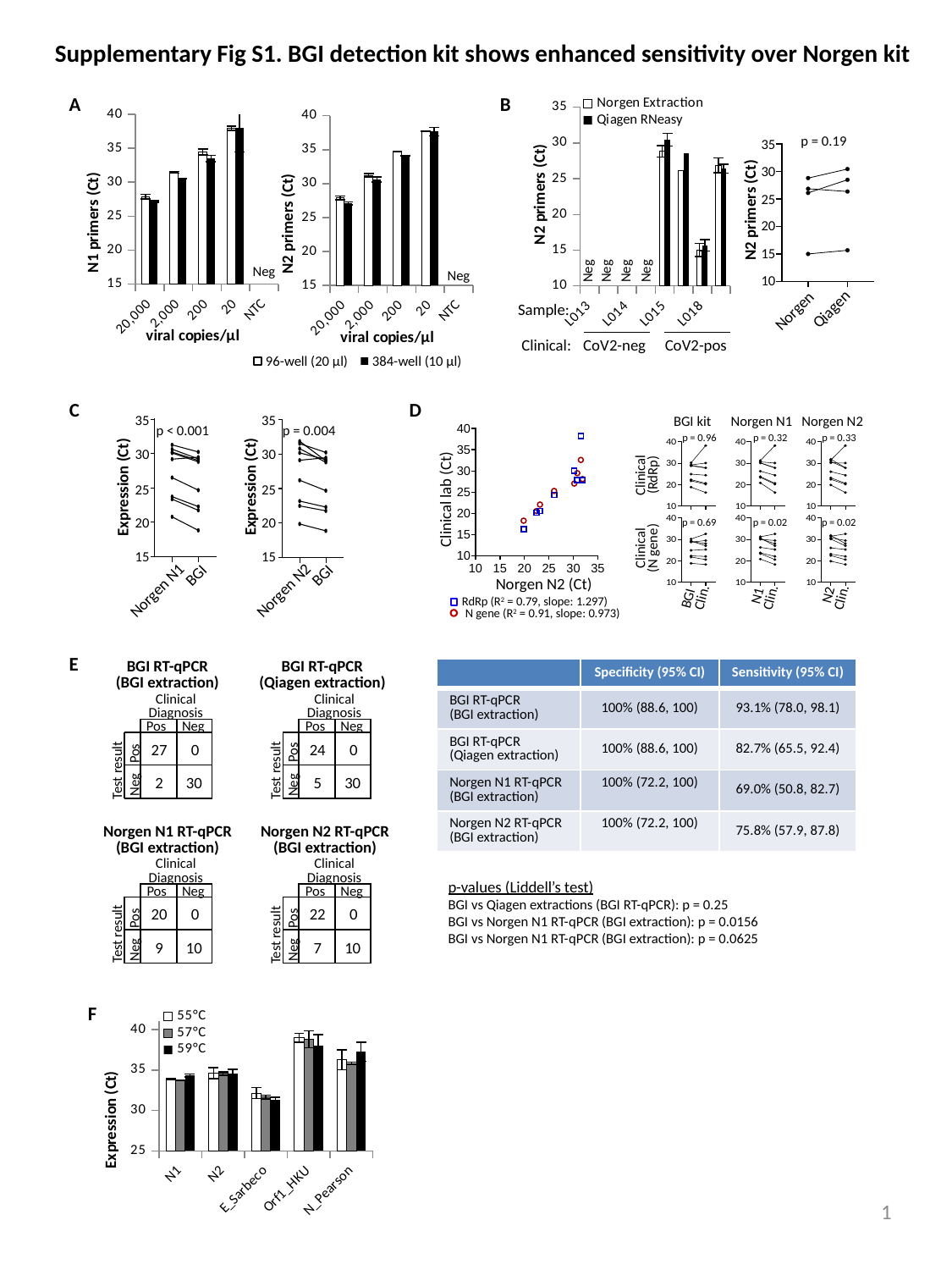

Supplementary Fig S1. BGI detection kit shows enhanced sensitivity over Norgen kit
A
B
### Chart
| Category | Norgen Extraction | Qiagen RNeasy |
|---|---|---|
| L013 | None | None |
| L013F | None | None |
| L014 | None | None |
| L017 | None | None |
| L015 | 28.83 | 30.46 |
| L016 | 26.14 | 28.51 |
| L018 | 15.025 | 15.695 |
| L019 | 26.88 | 26.384999999999998 |Neg
Neg
Neg
Neg
Sample:
CoV2-neg
CoV2-pos
Clinical:
### Chart
| Category | 96-well (20µl) | 384-well (10µl) |
|---|---|---|
| 20,000 | 27.91 | 27.16 |
| 2,000 | 31.455 | 30.51 |
| 200 | 34.445 | 33.519999999999996 |
| 20 | 37.965 | 38.055 |
| NTC | 0.0 | 0.0 |Neg
### Chart
| Category | 96-well (20µl) | 384-well (10µl) |
|---|---|---|
| 20,000 | 27.925 | 27.1 |
| 2,000 | 31.22 | 30.595 |
| 200 | 34.75 | 34.084999999999994 |
| 20 | 37.685 | 37.655 |
| NTC | 0.0 | 0.0 |Neg
384-well (10 µl)
96-well (20 µl)
p = 0.19
35
30
25
N2 primers (Ct)
20
15
10
Qiagen
Norgen
C
D
35
p < 0.001
30
Expression (Ct)
25
20
15
BGI
Norgen N1
35
p = 0.004
30
Expression (Ct)
25
20
15
BGI
Norgen N2
BGI kit
Norgen N1
Norgen N2
p = 0.96
p = 0.32
p = 0.33
 Clinical
(RdRp)
p = 0.69
p = 0.02
p = 0.02
 Clinical
(N gene)
N2
N1
Clin.
Clin.
Clin.
BGI
Clinical lab (Ct)
Norgen N2 (Ct)
RdRp (R2 = 0.79, slope: 1.297)
N gene (R2 = 0.91, slope: 0.973)
E
BGI RT-qPCR
(BGI extraction)
BGI RT-qPCR
(Qiagen extraction)
| | Specificity (95% CI) | Sensitivity (95% CI) |
| --- | --- | --- |
| BGI RT-qPCR (BGI extraction) | 100% (88.6, 100) | 93.1% (78.0, 98.1) |
| BGI RT-qPCR (Qiagen extraction) | 100% (88.6, 100) | 82.7% (65.5, 92.4) |
| Norgen N1 RT-qPCR (BGI extraction) | 100% (72.2, 100) | 69.0% (50.8, 82.7) |
| Norgen N2 RT-qPCR (BGI extraction) | 100% (72.2, 100) | 75.8% (57.9, 87.8) |
Clinical Diagnosis
Pos
Neg
27
0
Pos
Test result
2
30
Neg
Clinical Diagnosis
Pos
Neg
24
0
Pos
Test result
5
30
Neg
Norgen N1 RT-qPCR
(BGI extraction)
Norgen N2 RT-qPCR
(BGI extraction)
Clinical Diagnosis
Pos
Neg
20
0
Pos
Test result
9
10
Neg
Clinical Diagnosis
Pos
Neg
22
0
Pos
Test result
7
10
Neg
p-values (Liddell’s test)
BGI vs Qiagen extractions (BGI RT-qPCR): p = 0.25
BGI vs Norgen N1 RT-qPCR (BGI extraction): p = 0.0156
BGI vs Norgen N1 RT-qPCR (BGI extraction): p = 0.0625
F
### Chart
| Category | 55°C | 57°C | 59°C |
|---|---|---|---|
| N1 | 33.8830159585365 | 33.70430503750515 | 34.32147199843627 |
| N2 | 34.61145221839005 | 34.5593301913443 | 34.52674219955978 |
| E_Sarbeco | 32.15637254708245 | 31.669964167125976 | 31.287374374266975 |
| Orf1_HKU | 38.98872422795455 | 38.78352849000575 | 38.0365662828289 |
| N_Pearson | 36.277203171214325 | 35.80887213893938 | 37.250945284859725 |1

## Slide 2
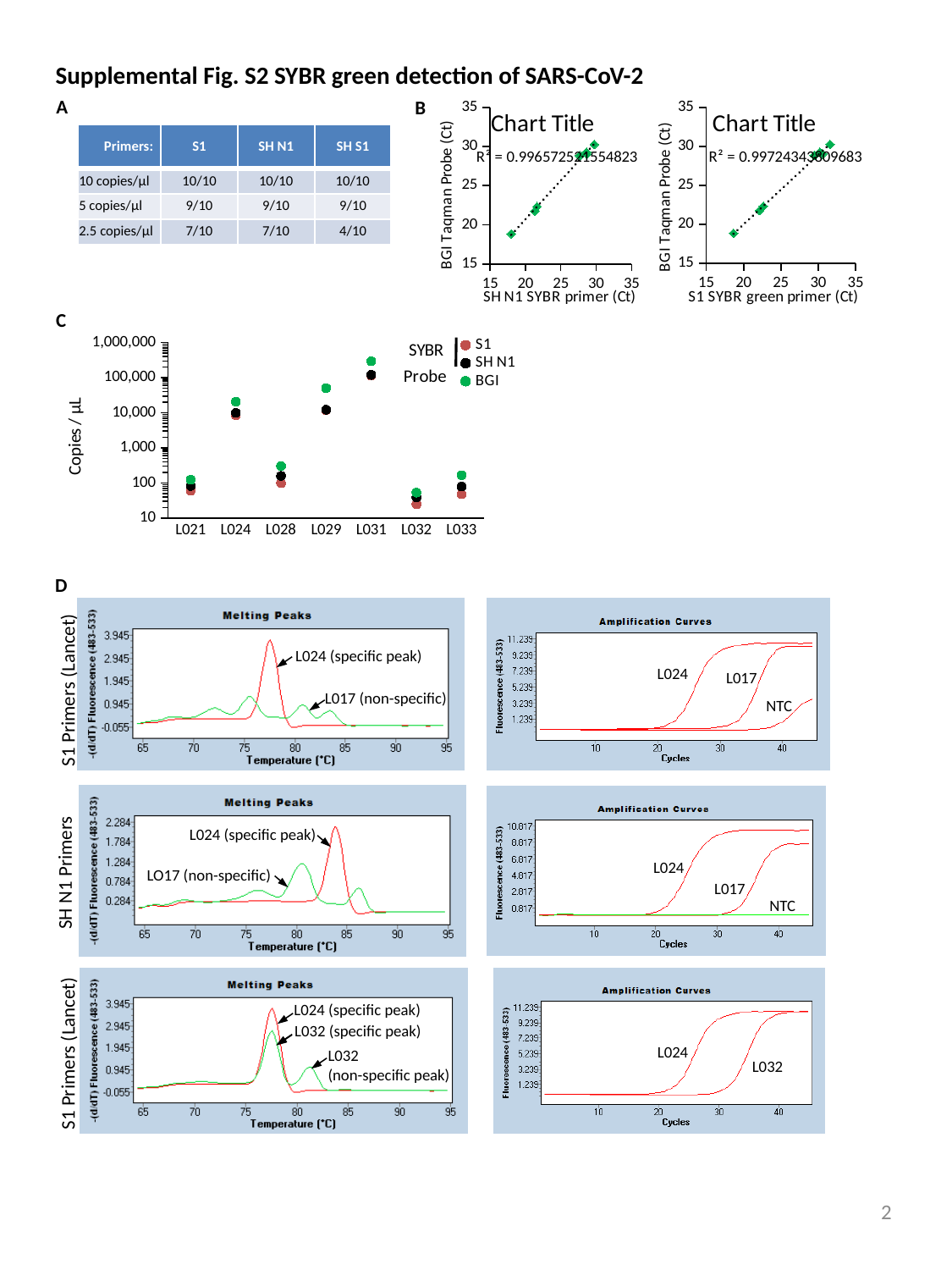

Supplemental Fig. S2 SYBR green detection of SARS-CoV-2
A
B
### Chart:
| Category | |
|---|---|
### Chart:
| Category | |
|---|---|| Primers: | S1 | SH N1 | SH S1 |
| --- | --- | --- | --- |
| 10 copies/μl | 10/10 | 10/10 | 10/10 |
| 5 copies/μl | 9/10 | 9/10 | 9/10 |
| 2.5 copies/μl | 7/10 | 7/10 | 4/10 |
### Chart
| Category | S1 | SH N1 | BGI |
|---|---|---|---|
| L021 | 60.0 | 81.6 | 123.0 |
| L024 | 8440.0 | 9880.0 | 20693.0 |
| L028 | 98.0 | 158.0 | 301.0 |
| L029 | 11880.0 | 12200.0 | 50504.0 |
| L031 | 114800.0 | 119600.0 | 291443.0 |
| L032 | 24.56 | 38.32 | 53.0 |
| L033 | 47.6 | 79.2 | 166.0 |C
D
L024 (specific peak)
L024
L017
S1 Primers (Lancet)
L017 (non-specific)
NTC
L024 (specific peak)
L024
SH N1 Primers
LO17 (non-specific)
L017
NTC
L024 (specific peak)
L032 (specific peak)
S1 Primers (Lancet)
L024
L032
(non-specific peak)
L032
2
